# Virtual Faces Expressing Emotions: An Initial Concomitant and Construct Validity Study

**DOI:** 10.3389/fnhum.2014.00787

**Published:** 2014-09-30

**Authors:** Christian C. Joyal, Laurence Jacob, Marie-Hélène Cigna, Jean-Pierre Guay, Patrice Renaud

**Affiliations:** ^1^Department of Psychology, University of Quebec at Trois-Rivières, Trois-Rivières, QC, Canada; ^2^Research Center, Philippe-Pinel Institute of Montreal, Montreal, QC, Canada; ^3^Department of Criminology, University of Montreal, Montreal, QC, Canada; ^4^Department of Psychology, University of Quebec in Outaouais, Gatineau, QC, Canada

**Keywords:** virtual, facial, expressions, emotions, validation

## Abstract

**Background:** Facial expressions of emotions represent classic stimuli for the study of social cognition. Developing virtual dynamic facial expressions of emotions, however, would open-up possibilities, both for fundamental and clinical research. For instance, virtual faces allow real-time Human–Computer retroactions between physiological measures and the virtual agent.

**Objectives:** The goal of this study was to initially assess concomitants and construct validity of a newly developed set of virtual faces expressing six fundamental emotions (happiness, surprise, anger, sadness, fear, and disgust). Recognition rates, facial electromyography (zygomatic major and corrugator supercilii muscles), and regional gaze fixation latencies (eyes and mouth regions) were compared in 41 adult volunteers (20 ♂, 21 ♀) during the presentation of video clips depicting real vs. virtual adults expressing emotions.

**Results:** Emotions expressed by each set of stimuli were similarly recognized, both by men and women. Accordingly, both sets of stimuli elicited similar activation of facial muscles and similar ocular fixation times in eye regions from man and woman participants.

**Conclusion:** Further validation studies can be performed with these virtual faces among clinical populations known to present social cognition difficulties. Brain–Computer Interface studies with feedback–feedforward interactions based on facial emotion expressions can also be conducted with these stimuli.

## Introduction

Recognizing emotions expressed non-verbally by others is crucial for harmonious interpersonal exchanges. A common approach to assess this capacity is the evaluation of facial expressions. Presentations of photographs of real faces allowed the classic discovery that humans are generally able to correctly perceive six fundamental emotions (happiness, surprise, fear, sadness, anger, and disgust) experienced by others from their facial expressions (Ekman and Oster, 1979). These stimuli also helped documenting social cognition impairment in neuropsychiatric disorders such as autism (e.g., Dapretto et al., 2006), schizophrenia (e.g., Kohler et al., 2010), and psychopathy (Deeley et al., 2006). Given their utility, a growing number of sets of facial stimuli were developed during the past decade, including the Montreal Set of Facial Displays of Emotion (Beaupré and Hess, 2005), the Karolinska Directed Emotional Faces (Goeleven et al., 2008), the NimStim set of facial expressions (Tottenham et al., 2009), the UC Davis set of emotion expressions (Tracy et al., 2009), the Radboud faces database (Langner et al., 2010), and the Umeå University database of facial expressions (Samuelsson et al., 2012). These sets, however, have limitations. First, they consist of static photographs of facial expressions from real persons, which cannot be readily modified to fit a specific requirement of particular studies (e.g., presenting elderly Caucasian females). Second, static facial stimuli elicit weaker muscle mimicry responses, and they are less ecologically valid than dynamic stimuli (Sato et al., 2008; Rymarczyk et al., 2011). Because recognition impairments encountered in clinical settings might be subtle, assessment of different emotional intensities is often required, which is better achieved with dynamic stimuli (incremental expression of emotions) than static photographs (Sato and Yoshikawa, 2007).

Custom-made video clips of human actors expressing emotions have also been used (Gosselin et al., 1995), although it is a time and financially consuming process. Recent sets of validated video clips are available (van der Schalk et al., 2011; Bänziger et al., 2012), but again, important factors such as personal expressive style and physical characteristics (facial physiognomy, eye–hair color, skin texture, etc.) of the stimuli are fixed and difficult to control. Furthermore, video clips are not ideal for novel treatment approaches that use Human–Computer Interfaces (HCI; Birbaumer et al., 2009; Renaud et al., 2010).

A promising avenue to address all these issues is the creation of virtual faces expressing emotions (Roesch et al., 2011). Animated synthetic faces expressing emotions allow controlling of a number of potential confounds (e.g., equivalent intensity, gaze, physical appearance, socio-demographic variables, head angle, ambient luminosity), while giving experimenters a tool to create specific stimuli corresponding to their particular demands. Before being used with HCI in research or clinical settings, sets of virtual faces expressing emotions must be validated. Although avatars expressing emotions are still rare (Krumhuber et al., 2012), interesting results emerged from previous studies. First, basic emotions are well recognized from simple computerized line drawing depicting facial muscle movements (Wehrle et al., 2000). Second, fundamental emotions expressed by synthetic faces are equally, if not better, recognized than those expressed by real persons (except maybe for disgust; Dyck et al., 2008). Third, virtual facial expressions of emotions elicit sub-cortical activation of equivalent magnitude than that observed with real facial expressions (Moser et al., 2007). Finally, clinical populations with deficits of social cognition also show impaired recognition of emotions expressed by avatars (Dyck et al., 2010). In brief, virtual faces expressing emotions represent a promising approach to evaluate aspects of social cognition both for fundamental and clinical research (Mühlberger et al., 2009).

We recently developed a set of adult (males and females) virtual faces from different ethnic backgrounds (Caucasian, African, Latin, or Asian), expressing seven facial emotional states (neutral, happiness, surprise, anger, sadness, fear, and disgust) with different intensities (40, 60, 100%), from different head angles (90°, 45°, and full frontal; Cigna et al., in press). The purpose of this study was to validate a dynamic version of these stimuli. In addition to verify convergent validity with stimuli of dynamic expressions from real persons, the goal of this study was to demonstrate construct validity with physiological measures traditionally associated with facial emotion recognition of human expressions: facial electromyography (fEMG) and eye-tracking.

Facial muscles of an observer generally react with congruent contractions while observing the face of a real human expressing a basic emotion (Dimberg, 1982). In particular, the zygomatic major (lip corner pulling movement) and corrugator supercilii (brow lowering movement) muscles are rapidly, unconsciously, and differentially activated following exposition to pictures of real faces expressing basic emotions (Dimberg and Thunberg, 1998; Dimberg et al., 2000). Traditionally, these muscles are used to distinguish between positive and negative emotional reactions (e.g., Cacioppo et al., 1986; Larsen et al., 2003). In psychiatry, fEMG have been used to demonstrate sub-activation of the zygomatic major and/or the corrugator supercilii muscles in autism (McIntosh et al., 2006), schizophrenia (Mattes et al., 1995), personality disorders (Herpertz et al., 2001), and conduct disorders (de Wied et al., 2006). Interestingly, virtual faces expressing basic emotions induce the same facial muscle activation in the observer as do real faces, with the same dynamic >static stimulus advantage (Weyers et al., 2006, 2009). Thus, recordings of the zygomatic major and the corrugator supercilii muscle activations should represent a good validity measure of computer-generated faces.

Eye-trackers are also useful in the study of visual emotion recognition because gaze fixations on critical facial areas (especially mouth and eyes) are associated with efficient judgment of facial expressions (Walker-Smith et al., 1977). As expected, different ocular scanning patterns and regional gaze fixations are found among persons with better (Hall et al., 2010) or poorer recognition of facial expressions of emotions (e.g., persons with autism, Dalton et al., 2005; schizophrenia, Loughland et al., 2002; or psychopathic traits, Dadds et al., 2008). During exposition to virtual expressions of emotions, very few eye-tracking studies are available, although the data seem comparable to those with real stimuli (e.g., Wieser et al., 2009). In brief, fEMG and eye-tracking measures could serve not only to validate virtual facial expressions of emotions, but also to demonstrate the possibility of using peripheral input (e.g., muscle activation and gaze fixations) with virtual stimuli for HCI. The main goal of this study was to conduct three types of validation with a new set of virtual faces expressing emotions: (1) primary (face) validity with recognition rates; (2) concurrent validity with another, validated instrument; and (3) criterion validity with facial muscle activation and eye gaze fixations. This study was based on three hypotheses. H1: the recognition rates would not differ significantly between the real and virtual conditions for any of the six expressed emotions; H2: real and virtual conditions would elicit similar mean activation of the zygomatic major and corrugator supercilii muscles for the six expressed emotions; H3: the mean time of gaze fixations on regions of interest would be similar in both conditions (real and virtual).

## Materials and Methods

### Participants

Forty-one adult Caucasian volunteers participated in the study (mean age: 24.7 ± 9.2, 18–60 interval; 20 males and 21 females). They were recruited via Facebook friends and university campus advertisement. Exclusion criteria were a history of epileptic seizures, having received a major mental disorder diagnosis, or suffering from motor impairment. Each participant signed an informed consent form and received a 10$ compensation for their collaboration. This number of participants was chosen based on previous studies concerned with emotional facial expressions of emotion (between 20 and 50 participants; Weyers et al., 2006, 2009; Dyck et al., 2008; Likowski et al., 2008; Mühlberger et al., 2009; Roesch et al., 2011; Krumhuber et al., 2012).

### Materials and measures

**Figure 1:**
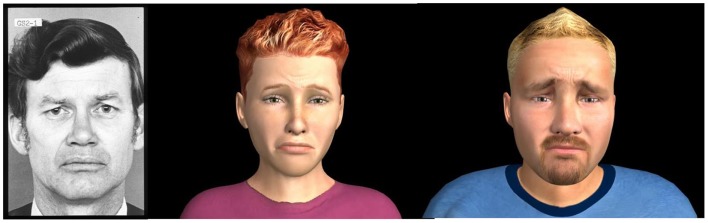
**Examples of 100% expression (sadness) by real and virtual stimuli are shown**.

**Figure 2:**
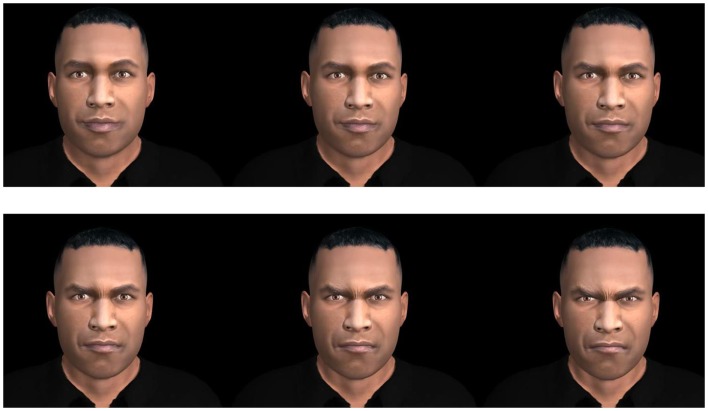
**Example of a sequence from neutral to 100% expression (anger) from a computer-generated face is shown**.

Participants were comfortably seated in front of a 19″ monitor in a sound attenuated, air-conditioned (19°C) laboratory room. The stimuli were video clips of real Caucasian adult faces and video clips of avatar Caucasian adult faces dynamically expressing a neutral state and the six basic emotions (happiness, surprise, anger, sadness, fear, and disgust). Video clips of real persons (one male, one female) were obtained from computerized morphing (FantaMorph software, Abrasoft) of two series of photographs from the classic Picture of Facial Affect set (Ekman and Friesen, 1976; from neutral to 100% intensity). Video clips of virtual faces were obtained from morphing (neutral to 100% intensity) static expressions of avatars from our newly developed set (one male, and female; Cigna et al., in press; Figure 1). The stimuli configurations were based on the POFA (Ekman and Friesen, 1976) and the descriptors of the Facial Action Coding System (Ekman et al., 2002). In collaboration with a professional computer graphic designer specialized in facial expressions (BehaVR solution)^1^, virtual dynamic facial movements were obtained by gradually moving multiple facial points (action units) along vectors involved in the 0–100% expressions (Rowland and Perrett, 1995). For the present study, 24 video clips were created: 2 (real and virtual) × 2 (man and woman) × 6 (emotions). A series example is depicted in Figure 2. Video clips of 2.5, 5, and 10 s. were obtained and pilot data indicated that 10 s presentations were optimal for eye-tracking analyses. Therefore, real and synthetic expressions were presented during 10 s, preceded by a 2 s central cross fixation. During the inter-stimulus intervals (max 10 s), participants had to select (mouse click) the emotion expressed by the stimulus from a multiple-choice questionnaire (Acrobat Pro software) appearing on the screen. Each stimulus was presented once, pseudo randomly, in four blocks of six emotions, counterbalanced across participants (Eyeworks presentation software, Eyetracking Inc., CA, USA).

**Figure 3:**
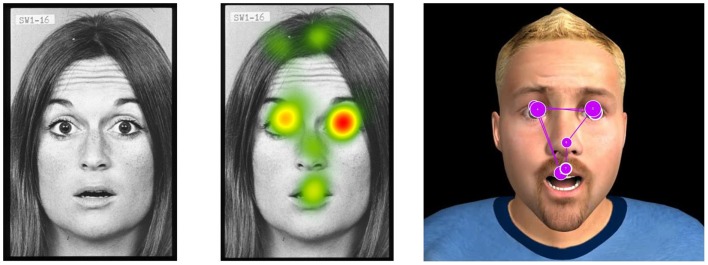
**Example of eye-tracking data (regional gaze fixations) on real and virtual stimuli is shown**.

Fiber contractions (microvolts) of the zygomatic major and the corrugator supercilii muscles (left side) were recorded with 7 mm bipolar (common mode rejection) Ag/AgCl pre-gelled adhesive electrodes^2^, placed in accordance with the guidelines of Fridlund and Cacioppo (1986). The skin was exfoliated with NuPrep (Weaver, USA) and cleansed with sterile alcohol prep pads (70%). The raw signal was pre-amplified through a MyoScan-Z sensor (Thought Technology, Montreal, QC, Canada) with built-in impedance check (<15 kΩ), referenced to the upper back. Data were relayed to a ProComp Infinity encoder (range of 0–2000 μV; Thought Technology) set at 2048 Hz, and post-processed with the Physiology Suite for Biograph Infinity (Thought Technology). Data were filtered with a 30 Hz high-pass filter, a 500 Hz low pass filter, and 60 Hz notch filter. Baseline EMG measures were obtained at the beginning of the session, during eye-tracking calibration. Gaze fixations were measured with a FaceLab5 eye-tracker (SeeingMachines, Australia), and regions of interest were defined as commissures of the eyes and the mouth (Eyeworks software; Figure 3). Assessments were completed in approximately 30 min.

### Statistical analyses

Emotion recognition and physiological data from each participant were recorded in Excel files and converted into SPSS for statistical analyses. First, recognition rates (%) for real vs. avatar stimuli from male and female participants were compared with Chi-square analyses, corrected (*p* < 0.008) and uncorrected (*p* < 0.05) for multiple comparisons. The main goal of this study was to demonstrate that the proportion of recognition of each expressed emotion would be statistically similar in both conditions (real vs. virtual). To this end, effect sizes (ES) were computed using the Cramer’s *V* statistic. Cramer’s *V* values of 0–10, 11–20, 21–30, and 31 and are considered null, small, medium, and large, respectively (Fox, 2009). Repeated measures analyses of variance (ANOVAs) between factors (real vs. virtual) with the within-subject factor emotion (happiness, surprise, anger, sadness, fear, or disgust) were also conducted on the mean fiber contractions of the zygomatic major and the corrugator supercilii muscles, as well as the mean time spent looking at the mouth, eye, and elsewhere. For these comparisons, ES were computed with the *r* formula, values of 0.10, 0.30, and 0.50 were considered small, medium, and large, respectively (Field, 2005).

### Ethical consideration

This study was approved by the ethical committee of the University of Quebec at Trois-Rivières (CER-12-186-06.09).

## Results

No significant difference emerged between male (90%) and female (92.1%) raters (data not shown). In accordance with H1, recognition rates of the whole sample did not differ significantly between real and virtual expressions, neither overall [90.4 vs. 91.7%, respectively; *X*^2^(1) = 0.07, *p* = 0.51] nor for each emotion (Table 1). ES was small between conditions for all emotions, including joy (0.10), surprise (0.08), anger (0.07), sadness (0.04), fear (0.12), and disgust (0.07) (Table 1). In accordance with H2, no difference emerged between the mean contractions of the zygomatic major or the corrugator supercilii muscles between both conditions for any emotions, with all ES below 0.19 (Table 2). Finally, in partial accordance with H3, only the time spent looking at the mouth differed significantly between conditions [Real > Virtual; *F*(1,29) = 3.84, *p* = 0.001, ES = 0.58; Table 3]. Overall, low ES demonstrate that very few difference exist between the real and virtual conditions. However, such low ES also generated weak statistical power (0.28 with an alpha set at 0.05 and 41 participants). Therefore, the possibility remains that these negative results reflect a type-II error (1 − power = 0.72).

**Table 1:** **Comparisons of recognition rates (%) between real and virtual facial expressions of emotions**.

	Real dynamic	Avatar dynamic	*X*^2^	*p*	ES
Overall	90.4	91.7	0.07	>0.5	0.03
Joy	98.9	86.7	0.16	>0.5	0.10
Surprise	97.6	91.5	0.53	>0.3	0.08
Anger	96.4	87.8	0.43	>0.5	0.07
Sadness	91.5	96.4	0.11	>0.5	0.04
Fear	85.3	92.7	1.1	>0.3	0.12
Disgust	70.8	98.8	0.35	>0.5	0.07

*ES, effect size (Cramer’s *V*)*.

**Table 2:** **Comparisons of mean (SD) facial muscle activations during presentations of real and virtual stimuli expressing the basic emotions**.

	Real (μV)	Avatar (μV)	*F*	*p*	ES
**ZYGOMATICUS MAJOR**					
Overall	4.05 (3.1)	3.89 (3.7)	0.40	>0.5	0.06
Joy	4.59 (4.6)	4.21 (4.1)	0.92	>0.3	0.14
Fear	4.61 (4.1)	4.31 (5.3)	0.49	>0.5	0.07
Anger	3.75 (3.1)	3.76 (3.9)	0.03	>0.5	0.00
Sadness	3.76 (3.4)	3.33 (3.5)	1.2	>0.25	0.18
Surprise	3.53 (3.0)	3.62 (3.9)	0.25	>0.5	0.04
Disgust	4.05 (3.5)	4.12 (4.9)	0.14	>0.5	0.02
**CORRUGATOR SUPERCILII**					
Overall	8.71 (4.8)	8.59 (5.1)	0.03	>0.5	0.05
Joy	9.43 (7.0)	7.58 (4.9)	0.89	>0.3	0.13
Fear	9.10 (4.9)	8.25 (4.7)	1.1	>0.3	0.17
Anger	8.68 (4.9)	11.27 (8.9)	0.86	>0.5	0.13
Sadness	8.62 (4.8)	8.44 (5.0)	0.39	>0.3	0.06
Surprise	8.38 (4.8)	8.14 (4.6)	0.55	>0.5	0.09
Disgust	8.07 (4.7)	7.89 (4.7)	0.38	>0.5	0.06

*ES, effect size (*r*)*.

**Table 3:** **Comparisons of mean (SD) duration (ms) of gaze fixations during presentations of real and virtual stimuli expressing the basic emotions**.

	Real (ms)	Avatar (ms)	*F*	*p*	ES
Eyes (overall)	4334.4 (1831)	4534.3 (1783)	0.94	>0.3	0.17
Joy	3511 (1211)	3792 (1488)	NS		
Fear	4333 (1682)	4594 (1822)	NS		
Anger	4752 (2100)	4983 (1923)	NS		
Sadness	5132 (2345)	5090 (2015)	NS		
Surprise	4289 (1819)	4499 (1885)	NS		
Disgust	3966 (1562)	4222 (1724)	NS		
Mouth (overall)	1075.1 (196)	811.1 (147)	3.84	0.001	0.58
Joy	1613 (257)	712 (123)	4.66	0.001	
Fear	977 (300)	821 (210)	NS		
Anger	869 (113)	776 (280)	NS		
Sadness	1015 (320)	935 (152)	NS		
Surprise	1165 (385)	906 (108)	NS		
Disgust	858 (104)	726 (225)	NS		
Elsewhere (over all)	4591.5 (395)	4651.6 (401)	0.29	>0.5	0.08

## Discussion

The main goal of this study was to initially assess concomitants and construct validity of computer-generated faces expressing emotions. No difference was found between recognition rates, facial muscle activation, and gaze time spent on the eye region of virtual and real facial expression of emotions. Thus, these virtual faces can be used for the study of facial emotion recognition. Basic emotions such as happiness, anger, fear, and sadness were all correctly recognized with rates higher than 80%, which is comparable to rates obtained with other virtual stimuli (Dyck et al., 2008; Krumhuber et al., 2012). Interestingly, disgust expressed by our avatars was correctly detected in 98% of the cases (compared with 71% for real stimuli), an improvement from older stimuli (Dyck et al., 2008; Krumhuber et al., 2012). The only difference we found between the real vs. virtual conditions was the time spent looking at the mouth region of the real stimuli, which might be due to an artifact. Our real stimuli were morphed photographs, which could introduce unnatural saccades or texture-smoothing from digital blending. In this study, for instance, the highest time spent looking at the mouth of real stimuli was associated with a jump in the smile of the female POFA picture set (abruptly showing her teeth). Thus, comparisons with video clips of real persons expressing emotions are warranted (van der Schalk et al., 2011). Still, these preliminary data are encouraging. They suggest that avatars could eventually serve alternative clinical approaches such as virtual reality immersion and HCI Birbaumer et al., 2009; Renaud et al., 2010). It could be hypothesized, for instance, that better detection of other’s facial expressions would be achieved through biofeedback based on facial EMG and avatars reacting with corresponding expressions (Allen et al., 2001; Bornemann et al., 2012).

Some limits associated with this study should be addressed by future investigation. First, as abovementioned, using video clips of real persons expressing emotions would be preferable to using morphed photographs. It would also allow presentation of colored stimuli in both conditions. Second, and most importantly, the small number of participants in the present study prevents demonstrating that the negative results were not due to a type-II statistical error related with a lack of power. Most studies using avatars expressing emotions are based on sample sized ranging from 20 to 50 participants (Weyers et al., 2006, 2009; Dyck et al., 2008; Likowski et al., 2008; Mühlberger et al., 2009; Roesch et al., 2011; Krumhuber et al., 2012), because recognition rates are elevated, physiological effects are strong, and effect sizes are high. Although demonstrating an absence of difference is more difficult, these and the present results suggest that no significant difference exist between recognition and reaction to real and virtual agent expression of emotions. Only the addition of more participants in future investigations with our avatars will allow discarding this possibility.

Finally, with the increasing availability of software enabling the creation of whole-body avatars (Renaud et al., 2014), these virtual faces could be used to assess and treat social cognition impairment in clinical settings. We truly believe that the future of social skill evaluation and training resides in virtual reality.

## Author Note

This study was presented in part at the 32nd annual meeting of the Association for Treatment of Sexual Abusers (ATSA), Chicago, 2013.

## Conflict of Interest Statement

The authors declare that the research was conducted in the absence of any commercial or financial relationships that could be construed as a potential conflict of interest.

## Footnotes

^1^http://www.behavrsolution.com

^2^http://www.bio-medical.com
